# Interleukin-8 and tumor necrosis factor-alpha in youth with mood disorders—A longitudinal study

**DOI:** 10.3389/fpsyt.2022.964538

**Published:** 2022-08-11

**Authors:** Maria Skibinska, Aleksandra Rajewska-Rager, Monika Dmitrzak-Weglarz, Pawel Kapelski, Natalia Lepczynska, Mariusz Kaczmarek, Joanna Pawlak

**Affiliations:** ^1^Protein Biomarkers Unit, Department of Psychiatric Genetics, Poznan University of Medical Sciences, Poznan, Poland; ^2^Department of Psychiatric Genetics, Poznan University of Medical Sciences, Poznan, Poland; ^3^Department of Child and Adolescent Psychiatry, Poznan University of Medical Sciences, Poznan, Poland; ^4^Department of Cancer Immunology, Poznan University of Medical Sciences, Poznan, Poland

**Keywords:** adolescent mood disorders, major depressive disorder (MDD), bipolar disorder (BD), cytokines, interleukin-8, tumor necrosis factor-alpha, biomarkers

## Abstract

Bipolar disorder (BD) is one of the most disabling psychiatric illnesses. Over half of BD patients experienced early onset of the disease, and in most cases, it begins with a depressed mood episode. Up to 50% of adolescents initially diagnosed with major depressive disorder (MDD) convert to bipolar spectrum disorder. Diagnostic tools or biomarkers to facilitate the prediction of diagnosis conversion from MDD to BD are still lacking. Our study aimed to find biomarkers of diagnosis conversion in young patients with mood disorders. We performed a 2-year follow-up study on 69 adolescent patients diagnosed with MDD or BD. The control group consisted of 31 healthy youths. We monitored diagnosis change from MDD to BD. Impulsiveness was assessed using Barratt Impulsiveness Scale (BIS-11) and defense mechanisms using Defense Style Questionnaire (DSQ-40). According to the immunological hypothesis of mood disorders, we investigated baseline cytokines levels either in depressive or hypomanic/manic episodes. We correlated interleukin 8 (IL-8) and Tumor Necrosis Factor-alpha (TNF-alpha) levels with clinical factors. We detected higher IL-8 and TNF-alpha in patients in hypomanic/manic compared to depressed episodes. We found correlations of cytokine levels with immature defense style. We did not discover predictors of diagnosis conversion from MDD to BD.

## Introduction

The prevalence of mood disorders worldwide was estimated to be 22%, with more than 1% bipolar disorder (BD) and 7% major depressive disorder (MDD) (1). Over 65% of BD patients experienced early onset of the disease: in most cases, it begins with a depressed mood episode (2). Up to 50% of adolescents initially diagnosed with MDD convert to bipolar spectrum disorder (3).

Early-onset BD is associated with a more severe disease course, psychotic features, mixed episodes, neurocognitive dysfunctions, frequent comorbidity of panic disorder, and psychoactive substance abuse (2). Increased risk of suicide in early-onset BD is reported (4). Differential diagnosis between MDD and BD remains a clinical challenge, especially in first- or recent-onset youth patients. Misdiagnosis of BD in the early course of the illness may result in inadequate medication, and hence more severe and lengthy episodes, a greater number of relapses and hospitalizations, and worse social functioning (5). Adequate treatment administered early may benefit a better prognosis for young patients with BD (6). Specific diagnostic tools or biomarkers to facilitate the prediction of diagnosis conversion from MDD to BD would be useful in clinical practice but are still unavailable (7).

Chronic inflammation has been implicated in psychiatric illnesses' pathophysiology. Extensive studies conducted in MDD and BD show increased levels of peripheral immune markers in patients with mood disorders (8). Dysregulation of pro-and anti-inflammatory cytokines during the first episodes and early disease stages of schizophrenia, BD, and MDD are reported (9–11), though studies were performed only on young adult groups. The objective, biological effect of cytokines on the pathophysiology of psychiatric disorders, including MDD and BD, may be confounded by mixed samples of adolescents and adults, particularly middle-aged people, with a first-episode of the illness (12).

Early-onset (before 18 years old) implicates a more severe genetic predisposition and disease course, making the adolescent group extremely valuable in studies on biological mechanisms contributing to mental illnesses. Signal detection may be enhanced among adolescents and young adults with bipolar disorder due to shorter illness duration and less medical comorbidity vs. middle-aged adults with bipolar disorder. Studies on inflammatory markers performed on homogenous adolescent groups with first- or recent-onset disease are scarce (13).

### Hypotheses

The study's primary aim was to conduct a comprehensive clinical assessment during a 2-year follow-up study in a group of adolescents and young adults with mood disorders: major depressive disorder (MDD) and bipolar disorder (BD).

The secondary aim was to find possible correlations between clinical factors and baseline serum IL1-beta, IL-2, IL-4, IL-5, IL-6, IL-8, IL-10, IL-12 p70, IFN-gamma, and TNF-alpha levels to find predictive markers of disease progression and diagnosis conversion from MDD to BD in young patients with clinical variables (impulsivity and defense mechanisms).

We hypothesized that:

(1) There are differences in baseline serum cytokine levels between major depressive disorder (MDD), bipolar disorder (BD) patients, and controls. (2) Baseline cytokine levels may predict diagnosis change from MDD to BD in young patients. (3) Baseline cytokine concentrations correlate to clinical factors (medication status, family history of affective disorders, severity of depressive and manic symptoms, or gender). (4) Baseline cytokine levels correlate with impulsivity (measured using Barratt Impulsiveness Scale, BIS-11) and with defense mechanisms (measured using Defense Style Questionnaire, DSQ-40).

## Materials and methods

### Participants

In a 2-year follow-up study, we involved 69 patients, with a mean age of 18.49 (±3.2), with a diagnosis of mood disorders: major depressive disorder (MDD; *n* = 44) or bipolar disorder (BD; *n* = 25). Research participants were in- or out-patients from: Child and Adolescent Psychiatry or the Adult Psychiatry Ward, and the Outpatients Clinic at the Department of Psychiatry, Poznan University of Medical Sciences, Poland. Lifetime diagnosis and current mental status were assessed by experienced psychiatrists. Clinical evaluation was carried out across five visits during a 2-year follow-up: the inclusion in the study (baseline visit), then after three, 6 months, 1 year, and 2 years. The diagnosis was verified at each time-point by two independent psychiatrists, according to ICD-10 and DSM-IV criteria using standard instrumentation to confirm the diagnosis in adolescent patients: The Kiddie Schedule for Affective Disorders—Present and Lifetime Version (KSADS-PL) (14), and in adult patients: Structured Clinical Interview for DSM-IV (SCID) (15).

The severity of mood symptoms was evaluated using the Hamilton Depression Rating Scale (HDRS-17) (16) and the Young Mania Rating Scale (YMRS) (17). The cutoff points for depressed mood were HDRS-17 ratings ≥ 8 and for hypomanic/manic YMRS ≥ 12.

To observe diagnosis conversion, we monitored MDD patients group for any diagnosis change or conversion to BP in 2 years of observation.

All participants completed the self-report Defense Style Questionnaire (DSQ-40). Forty questions were grouped in 20 individual defenses covered three different defense styles: Mature (composed of: anticipation, humor, suppression and sublimation); Neurotic (encompassing: pseudo-altruism, idealization, reaction formation, undoing), and Immature (consisting of: acting out, denial, devaluation, displacement, dissociation, autistic fantasy, isolation, passive aggression, projection, rationalization, somatization, splitting) mechanisms (18).

The impulsiveness assessment was conducted using the self-reporting Barratt Impulsiveness Scale (BIS-11). Six correlated first-order constructs (attention, cognitive instability, motor, perseverance, self-control, cognitive complexity) and three second-order factors (attention, motor and non-planning) were analyzed (19, 20).

The Ethics Committee at the Poznan University of Medical Sciences approved the study protocol (permission no. 362/11) in accordance with the Declaration of Helsinki. All study participants were Caucasian and of Polish origin, from the Greater Poland region. Written informed consent to participate in the study was obtained from the subjects or their legal guardians before the commencement of the study. The exclusion criteria were: any severe medical or neurological illness, intellectual disability disorder, a pervasive developmental disorder, pregnancy.

The control group consisted of 31 healthy persons, with a mean age of 21.15 (±2.68). Control participants underwent psychiatric examination. Exclusion criteria were: any psychiatric diagnosis, family history of severe psychiatric disorders, substance abuse, or severe medical problems.

### Serum cytokine determination

Ten milliliter of venous blood was withdrawn into anticoagulant-free tubes between 7.30 and 9.30 h after overnight fasting. After 1 h incubation, the serum was separated by centrifugation, aliquoted, and stored at −70 until analysis. Human High Sensitivity Magnetic Luminex Performance Assay (R&D) was used in cytokine: IL1-beta, IL-2, IL-4, IL-5, IL-6, IL-8, IL-10, IL-12 p70, IFN-gamma, TNF-alpha serum levels determination. Serum samples, after thawing, were centrifuged at 16,000 g for 4 min before analysis. Quality controls of high, medium and low analyte concentration [Luminex Performance Assay Control Set 889 (Ref QC11)] were run on each plate. All samples were diluted 1:2 in Calibrator Diluent to a final volume of 100 μL, according to the manufacturer's instructions. Plates were incubated overnight at 4 degrees on a shaker set at 800 rpm. All samples and standards were run in duplicates. Detection steps were performed strictly in accordance with the manufacturer's procedures. All plates were run in one batch, on the same kit lot#, by the same experienced operator. Standard curves range: IL1-beta 1,400–0.34 pg/ml, IL-2 2,500–0.61 pg/ml, IL-4 7,100–1.73 pg/ml, IL-5 1,400–0.34 pg/ml, IL-6 4,000–0.89 pg/ml, IL-8 3,200–0.78 pg/ml, IL-10 1,500–0.37 pg/ml, IL-12 p70 25,500–6.23 pg/ml, IFN-gamma 20,000–4.88 pg/ml, TNF-alpha 3,200–0.78 pg/ml. Intra-assay and inter-assay variability was <5 and <10% coefficient of variation (CV) (accordingly).

### Statistics

The normality of the data was checked using the Kolmogorov-Smirnoff test. Non-parametric tests: Mann-Whitney *U*-test (for two-group comparisons) and Spearman's correlation were applied in analyses. The General Linear Model (GLM) correction for age and sex was applied. *Post-hoc* power analysis for significant results was performed using the G^*^Power program. Analyses were conducted using the Statistica v13 programme.

## Results

### Clinical characteristics of the groups studied

Forty-four patients with major depressive disorder (MDD) and twenty-five patients with bipolar disorder (BD) in hypomania/mania (*n* = 15) or mixed (*n* = 10) episodes were recruited for the study. In this group during follow-up visits 34 patients with MDD and 13 patients with BD achieved full remission (euthymia). Twelve patients (27.3%) of the MDD group converted their diagnosis to BD during a 2-year observation of clinical status. The drop-out rate was 25% of patients.

In MDD as well as in the BD subgroup, there was a significant improvement in HDRS-17 (*p* < 0.01) or YMRS (*p* < 0.01) scores (respectively) during treatment.

At the baseline visit, 16 patients (36%) with MDD and four patients (16%) with BD were drug-free, and there was a statistical trend (*p* = 0.07) in the difference of the number of drug-free patients between the MDD and BD group.

Forty-nine patients medicated before entering the study were treated with: antidepressants (*n* = 22), neuroleptics (*n* = 24), benzodiazepines (*n* = 2) or mood stabilizers (*n* = 16) either in monotherapy (*n* = 29) or polytherapy (*n* = 20). Depending on the current mood episode, all patients received pharmacological treatment (SSRI or TCA) or mood stabilizers after inclusion in the study. Demographic data are presented in Table 1.

**Table 1:** Clinical characteristics of the study group at baseline.

	**All patients**	**MDD**	**BD**	**HC**
*n*	69	44	25	31
Female/male	51/18	35/9	16/9	26/5
Mean age	18.49 (±3.2)	18.61 (±3.42)	18.28 (±2.82)	21.1 (± 2.76)
Mean age at illness onset	16.48 (±2.37)	16.42 (±2.4)	16.61 (±2.35)	NA
Drug free yes/no	20/49	16/28	4/21	
Inpatient/ outpatient	55/14	32/12	23/2	
Mean number of hospitalizations	1.29 (±0.78)	1.3 (±0.86)	1.26 (±0.62)	
Family history of bipolar disorder yes/no	34/35	26/18	8/17	
HDRS-17	14.33 (±8.18)	19.25 (±5.19)	5.68 (±4.37)	
YMRS	7.11 (±9.49)	0.95 (±1.57)	17.96 (±7.67)	
IL-8 (pg/mL), median (IQR)	7.84 (5.79–12.72)	7.4 (5.37–11.36)	10.89 (7.39–17.81)	10.22 (7.49–14.03)
TNF–alpha (pg/mL), median (IQR)	6.95 (5.56–9.28)	6.58 (5.29–7.91)	8.52 (6.14–10.01)	6.21 (5.3–7.23)

MDD, Major Depressive Disorder; BD, Bipolar Disorder; HC, Healthy Controls; HDRS-17, HamILton Depression Rating Scale (17-item); YMRS, Young Mania Rating Scale; IL-8, interleukin 8; TNF-alpha, tumor necrosis factor alpha; IQR, interquartile range.

### Serum cytokine detection

Out of 10 cytokines: IL1-beta, IL-2, IL-4, IL-5, IL-6, IL-8, IL-10, IL-12 p70, IFN-gamma, TNF-alpha analyzed in sera using High Sensitivity Luminex Assay, only IL-8 and TNF-alpha reached detectable levels in over 80% of samples. IL1-beta, IL-2, IL-4, IL-5, IL-6, IL-10, IL-12 p70, IFN-gamma concentrations in most samples were under the detection limit of the assay used, thus we excluded them from the statistical analyses.

### Comparisons of IL-8 and TNF-alpha levels

Comparing baseline cytokine levels in the whole group of patients (MDD + BD) with healthy controls (HC), we did not find differences for IL-8 (*p* = 0.19) or TNF-alpha (*p* = 0.087) concentrations. We detected a significantly lower baseline IL-8 level in MDD patients (*p* = 0.026, power 0.8) and a higher TNF-alpha level in BD patients (*p* = 0.004, power 0.85) compared to HC.

We detected higher concentrations of IL-8 (*p* = 0.033, power 0.6) and TNF-alpha (*p* = 0.022, power 0.64) in BD patients, compared to MDD subjects.

We did not observe any differences between the patient groups divided with regard to diagnosis conversion, observed after a 2-year follow-up (IL-8 *p* = 0.6, TNF-alpha *p* = 0.8), and considering any family history of affective disorders (IL-8 *p* = 0.13, TNF-alpha *p* = 0.22). A statistical trend toward significance (*p* = 0.06) of lower IL-8 in drug-free patients, and no differences in TNF-alpha (*p* = 0.4) levels with regard to medication status were detected. The results are presented in Table 2.

**Table 2:** Baseline comparisons of interleukin 8 (IL-8) and tumor necrosis factor alpha (TNF-alpha) levels with regard to clinical factors.

**Group**	**Cytokine**	***Z***	***P***
MDD+BD *vs*. HC	IL-8	−1.323	0.186
	TNF-alpha	1.709	0.087
MDD *vs*. HC	IL-8	−2.233	**0.026**
	TNF-alpha	0.559	0.576
BD *vs*. HC	IL-8	0.486	0.627
	TNF-alpha	2.884	**0.004**
MDD *vs*. BD	IL-8	−2.135	**0.033**
	TNF-alpha	−2.283	**0.022**
Diagnosis conversion from MDD to BD	IL-8	0.514	0.607
	TNF-alpha	−0.284	0.776
Family history of affective disorders	IL-8	−1.512	0.130
	TNF-alpha	−1.233	0.217
Drug-free *vs*. medicated	IL-8	1.851	0.064
	TNF-alpha	0.820	0.412

Mann-Whitney U-test; MDD, Major Depressive Disorder; BD, Bipolar Disorder; HC, Healthy Controls. Bold values means statistical significance p < 0.05.

A correction for age and gender revealed the influence of age on IL-8 levels (*p* = 0.008) in MDD group. No other dependencies with regards to age were noticed for IL-8 levels in BD (*p* = 0.06) and controls (*p* = 0.086), nor for TNF-alpha (MDD *p* = 0.06; BD *p* = 0.96; HC *p* = 0.11). Gender did not influence IL-8 and TNF-alpha levels in any of the subgroups.

### The correlation between IL-8, TNF-alpha, and symptoms severity

We did not find any dependence of baseline HDRS-17 scores with IL-8 (*p* = 0.44) nor TNF-alpha (*p* = 0.39) in MDD patients. In the BD subgroup we did not observe any correlation of YMRS scores either with IL-8 (*p* = 0.78) or TNF-alpha (*p* = 0.6).

### Correlations between IL-8, TNF-alpha, defense mechanisms (DSQ-40) and impulsivity (BIS-11)

#### DSQ-40

In the MDD group we detected significant correlations between IL-8 and: autistic fantasy (*R* = −0.45, *p* = 0.002), somatization (*R* = −0.42, *p* = 0.005), and immature subdimension (*R* = −0.3, *p* = 0.049). TNF-alpha correlated with autistic fantasy (*R* = −0.37, *p* = 0.01).

In the BD group we found correlations between IL-8 and: pseudoaltruism (*R* = 0.47, *p* = 0.02), isolation (*R* = −0.51, *p* = 0.01), and mature subdimension (*R* = 0.49, *p* = 0.02), while TNF-alpha correlated with isolation (*R* = 0.48, *p* = 0.02).

In the HC group we found a correlation between IL-8 and pseudoagression (*R* = 0.36, *p* = 0.05).

#### BIS-11

We detected correlations between IL-8 and cognitive instability (*R* = 0.46, *p* = 0.03) in BD patients, and between TNF-alpha and self-control (*R* = −0.37, *p* = 0.047) in the HC group, using Spearman's correlation.

The results of Spearman's correlation analysis are presented in Supplementary Table 1.

## Discussion

Studies of cytokine levels in adolescent mood disorders are scarce. Our study aimed to find biological and clinical predictors of diagnosis conversion from major depressive disorder (MDD) to bipolar disorder (BD) in a prospective study of adolescent and young adult patients with mood disorders. Baseline cytokine levels and clinical factors were compared concerning final diagnosis after a 2-year observation. To the best of our knowledge, the presented research is the first longitudinal study analyzing biological and clinical factors conducted in youth patients, focused on diagnosis conversion to BD.

We noticed a higher rate (27.3%) of diagnosis conversion in MDD patients during 2 years of follow-up comparing to the study by James et al. (21) in a cohort of young people in England, the only one up to date performed on adolescents. The overall conversion rate to BD was 5.6% in a minimum of 4-year (range 4–12 years) follow-up period, with greater conversion rates in female patients and psychotic depression (21). A higher rate of diagnosis conversion in our MDD group might be attributable to the prospective nature of the study design, with a focus on the hypomanic/manic symptoms tracking during four follow-up visits. In the retrospective study on Polish adult patients (*n* = 157) diagnosis conversion was noticed in the 32.8% of the total sample. Almost half of the conversions occurred within the first 5 years of the MDD diagnosis (5). In the two most recent population-based studies conducted in Finland and Korea, conversion rates from MDD to BD were: 11.1% in 15-year observation (22), and 6.5% in 8-year follow-up (23), respectively.

Our study revealed significantly lower IL-8 and TNF-alpha levels in depressed patients compared to hypomanic/manic adolescents and young adults with mood disorders. Compared to healthy controls, lower IL-8 levels in depressed patients and higher TNF-alpha levels in hypomanic/manic patients were detected. No differences in IL-8 and TNF-alpha levels in patient groups divided with regard to diagnosis conversion to BD, family history of affective disorders, and medication status were observed. We detected significant correlations between IL-8 and TNF-alpha levels and defense mechanisms.

According to the neuroimmunological hypothesis of affective disorders, numerous studies on circulating cytokines in major depressive disorder and bipolar disorder have been conducted, resulting in meta-analyses confirming alterations in pro- and anti-inflammatory cytokine levels (24–29). Most of the studies were performed on adult groups: only one recent meta-analysis by D'Acunto et al. (25) was performed for studies on cytokine levels in children and adolescents with MDD. Only five studies were included in the quantitative synthesis. TNF-alpha was found to be higher in depressed participants, while no differences were detected for IFN-gamma, IL1-beta, IL-4, IL-6, IL-8, and IL-10 (25). Results of our study are not consistent with abovementioned meta-analysis, with lower IL-8 level in the subgroup with MDD compared to healthy controls, while TNF-alpha showed no differences between MDD and the HC group.

The most recent and largest meta-analysis on circulating inflammatory factors in BD was conducted by Solmi et al. (27), including 49 studies on adult patient groups. Analyzing results separately for depression, mania, and mixed state, decreased TNF-alpha levels were found for each mood state (27). Luo et al. (30) compared TNF-alpha levels in depressed, manic and mixed adult patients with BD. Similarly to our results, higher TNF-alpha levels in manic and lower TNF-alpha levels in depressed patients were detected (30). In contrast, we found significantly elevated TNF-alpha levels in the hypomania/mania youth subgroup compared to healthy controls.

Interleukin 8 (IL-8), a pro-inflammatory cytokine in the brain, is synthesized mainly by microglia and astrocytes. IL-8 is produced early in the inflammatory response, persisting for days or weeks, acting in the endocrine manner (31). Thus, IL-8 might be specific for chronic inflammatory changes observed in neuropsychiatric disorders (32). Meta-analyses show a lack of differences in circulating IL-8 levels between adult MDD patients and healthy controls (26, 33, 34). Our results oppose those obtained in most studies conducted on depressed adult patients. We detected decreased IL-8 serum levels in youth MDD patients compared to the HC group. In a recent meta-analysis, Cakici et al. showed significantly reduced IL-8 levels in drug-naïve first-episode MDD patients (10). The MDD group in our study, though not fully drug-naïve due to recent disease onset, was not medicated for a prolonged time, thus corresponding more to the drug-naïve than chronically medicated patients.

There are only a few studies on inflammatory markers in adolescent BD patients. Two reports by Goldstein et al. indicate the significant correlation between pro-inflammatory IL-6 and hsCRP levels with mood symptoms, suicidal behavior, and illness duration (13, 35). Increased pro-inflammatory cytokines IL-6 and TNF-alpha were reported in the adolescent BD group compared with healthy controls and increased cardiovascular risk (36). The most recent prospective study by Karthikeyan et al. showed increased levels of hsCRP and pro- to anti-inflammatory ratios in the exacerbation of mood symptoms (37). Other studies did not confirm any differences in inflammatory markers in youth patients with BD (38, 39). Wu et al. (40) in the retrospective study on a large cohort of MDD (*n* = 160) and BD (*n* = 101) adolescents (aged 10–18 years) performed discriminative analysis based on the results of routine laboratory tests combining hematopoietic and immune-inflammatory systems, liver functions, metabolism, and hormones. Age, direct bilirubin (DBIL), lactic dehydrogenase (LDH), CRP, and free triiodothyronine (FT3) were finally included in the model, with well external validation (AUC = 0.714). Age, LDH, and FT3 were negatively, while DBIL positively correlated with BD diagnosis. Regrettably, diagnosis conversion in MDD group was not monitored in this study (40).

A relationship between defense mechanisms and bipolar disorder has been found (41), but there is a gap in the studies on the biological mechanisms. Our study searched for the relationship between inflammatory factors, impulsivity, defense mechanisms, and mood disorders in youth patients. To the best of our knowledge, we present the first study correlating cytokine levels with impulsiveness (Barratt Impulsiveness Scale, BIS-11) and defense mechanisms (Defense Style Questionnaire, DSQ-40) scores in BD and MDD. Our results showed a negative correlation of immature defense mechanisms and pro-inflammatory cytokines (IL-8 and TNF-alpha). Lower levels of IL-8 and TNF-alpha in MDD correlated with higher scores on the Immature factor and its particular components: autistic fantasy and somatization (only IL-8). In BD patients, both studied cytokine levels correlated negatively with higher isolation scores. The use of immature defense mechanisms is characteristic of patients with mental disorders, including MDD and BD. Wang et al. found positive correlation between childhood trauma and immature defense mechanisms in adult BD patients (42). Childhood trauma is also associated with increased inflammatory response (43, 44). Although our study did not consider childhood trauma, it may be one of the factors associated with immature defense mechanisms and inflammatory processes. This result needs to be replicated on a larger group regarding the small BD sample size and relatively poor *post-hoc* power. We hypothesized that higher inflammation would correlate with immature and neurotic defense mechanisms (which are connected to clinical features of BD). Unexpectedly, our results are opposite to our hypothesis. This negative correlation of pro-inflammatory cytokines with immature defense styles might indicate compensatory mechanisms participating in biological processes underlying the regulation of psychological defense mechanisms.

We did not find any correlations between IL-8 and TNF-alpha levels with impulsivity either in MDD or BD patients in our study. We detected a significant negative correlation between TNF-alpha levels and the self-control dimension of the BIS-11 scale only in healthy controls. Impulsivity is a crucial element of suicidality, which increases mortality rates in psychiatric diseases. Only several studies report the correlation between inflammatory markers and impulsive and aggressive behavior (45–47). Isung et al. found higher concentrations of interleukin 6 in suicide attempters (48). None of the abovementioned studies used the BIS-11 scale in assessing impulsivity scores.

## Conclusions

Our study revealed alterations in TNF-alpha and IL-8 levels in youth with mood disorders. Both cytokine levels were found to be lowered in depressed patients compare to hypomanic/manic patients. Negative correlations between TNF-alpha, IL-8 levels and immature defense mechanisms in MDD and BD were found. These results need replication because this is the first study that correlates cytokine levels with comprehensive clinical evaluation in adolescents with mood disorders.

## Limitations

The limitations of this study are a small sample size, a lack of an exact age-matched control group, a high drop-out rate during the 2-year clinical observation of disease progression. Studies with larger samples and a more prolonged clinical observation period are necessary. Environmental stressors such as abuse at home or bullying at school, which may influence cytokine expression, were not monitored. Although High Sensitivity Magnetic Luminex Assay was used to analyze cytokine levels in the participant's sera, we could detect only IL-8 and TNF-alpha with satisfactory performance. IL1-beta, IL-2, IL-4, IL-5, IL-6, IL-10, IL-12 p70, IFN-gamma levels were under the assay's detection limit in most samples or had threshold values with high CV between duplicates, thus making results not precise.

## Data availability statement

The raw data supporting the conclusions of this article will be made available by the authors, without undue reservation.

## Ethics statement

The studies involving human participants were reviewed and approved by the Bioethics Committee, Poznan University of Medical Sciences, Poland, permission no. 362/11. Written informed consent to participate in this study was provided by the participants' legal guardian/next of kin.

## Author contributions

AR-R and MS: conceptualization, methodology, and project administration. MS: software, formal analysis, resources, writing—original draft preparation, and visualization. MK, MD-W, and JP: validation. AR-R, PK, NL, and JP: investigation. MD-W: data curation. AR-R: writing—review and editing and funding acquisition. JP: supervision. All authors have read and agreed to the published version of the manuscript.

## Funding

This work was supported by the National Science Centre in Poland no. UMO-2011/03/D/NZ5/06146 and statute sources of the Department of Psychiatric Genetics, PUMS no. 502-20-22196440. Funding agents did not influence the study design, patient's recruitment, analyses, interpretation of the results, and manuscript preparation.

## Conflict of interest

The authors declare that the research was conducted in the absence of any commercial or financial relationships that could be construed as a potential conflict of interest.

## Publisher's note

All claims expressed in this article are solely those of the authors and do not necessarily represent those of their affiliated organizations, or those of the publisher, the editors and the reviewers. Any product that may be evaluated in this article, or claim that may be made by its manufacturer, is not guaranteed or endorsed by the publisher.
